# PennDemic Simulation Framework: An Innovative Approach to Increase Student Interest and Confidence in Disasters Preparedness/Response and Interdisciplinary Teamwork

**DOI:** 10.3389/fpubh.2021.682112

**Published:** 2021-05-28

**Authors:** Stephen D. Cole, Hillary C. M. Nelson, Bonnie D. Jenkins, Cathy Y. Poon, Shelley C. Rankin, Deborah E. Becker

**Affiliations:** ^1^Department of Pathobiology, School of Veterinary Medicine, University of Pennsylvania, Philadelphia, PA, United States; ^2^Master of Public Health Program, Department of Biochemistry and Biophysics, Perelman School of Medicine, University of Pennsylvania, Philadelphia, PA, United States; ^3^School of Foreign Service, Georgetown University, Washington, DC, United States; ^4^Elliott School of International Affairs, George Washington University, Washington, DC, United States; ^5^Brookings Institute, Washington, DC, United States; ^6^Department of Pharmacy Practice and Pharmacy Administration, College of Pharmacy, University of the Sciences, Philadelphia, PA, United States; ^7^Department of Biobehavioral Health Sciences, School of Nursing, University of Pennsylvania, Philadelphia, PA, United States

**Keywords:** interprofessional education, disaster, simulation, infectious disease, interdisciplinary teamwork

## Abstract

An interdisciplinary group from two higher-education institutions in Philadelphia developed a novel framework for interprofessional education. This framework was applied to two different scenarios disease outbreak and natural disaster, which were used in simulations in 2018 and 2020. By design, these simulations included students from a broad range of disciplines, beyond the typical healthcare fields. Students were first grouped by discipline and were then placed in interdisciplinary teams for the rest of the scenario. Students were administered four surveys throughout which included 10 point-Likert scale and free response items. A statistically significant post-simulation increase in student interest and confidence was found. Survey analysis also revealed higher scores of positive group behaviors among interdisciplinary teams when compared to discipline groups. Importantly, students realized the importance of broad representation of disciplines for disaster preparedness. The PennDemic framework may be helpful for teams looking to develop simulations to build interest and confidence in disaster preparedness/response and interdisciplinary teamwork.

## Introduction

Response to any disaster (i.e., infectious disease outbreak or pandemic, natural disaster, radiologic, or chemical event) is multifaceted and complex, and it requires the involvement of individuals trained across a variety of disciplines (1, 2). The type of disaster, the spectrum of organizations, agencies, and stakeholders often vary, but the same non-technical social or cognitive skills (“soft skills”) may apply (3–5). The ability to facilitate collaboration and to work across fields is a critical tool that has been highlighted in disaster response competencies and non-technical skill sets developed by several groups (5–7).

Educational training in disaster preparedness has typically engaged healthcare students in medicine, nursing, and pharmacy (8, 9). Multiple studies have shown that interprofessional disaster simulation scenarios can be effective tools to build confidence and readiness in disaster response (10–13). Healthcare education has embraced the need for interprofessional education (IPE) and Interprofessional Education Collaborative (IPEC) core competencies are now found in many healthcare disciplines (14). Public health is the latest field to require IPE as a foundational competency for all accredited programs (15). However, few IPE activities have included non-healthcare professions and disciplines such as public health, let alone other critical fields like veterinary medicine, social work, counseling, design, basic science, or engineering (16, 17), even though these groups might add useful perspective and significant expertise to a response.

## Pedagogical Framework

In 2018, colleagues at the University of Pennsylvania decided to develop an interdisciplinary disaster simulation that would engage graduate students from a broad range of disciplines across the entire institution. The institution is well-suited for an interdisciplinary disaster simulation because of the diversity of programs that are all situated on a single campus. In addition, there are well-developed ties with the University of the Sciences, a neighboring institution with complementary programs.

**Figure 1:**
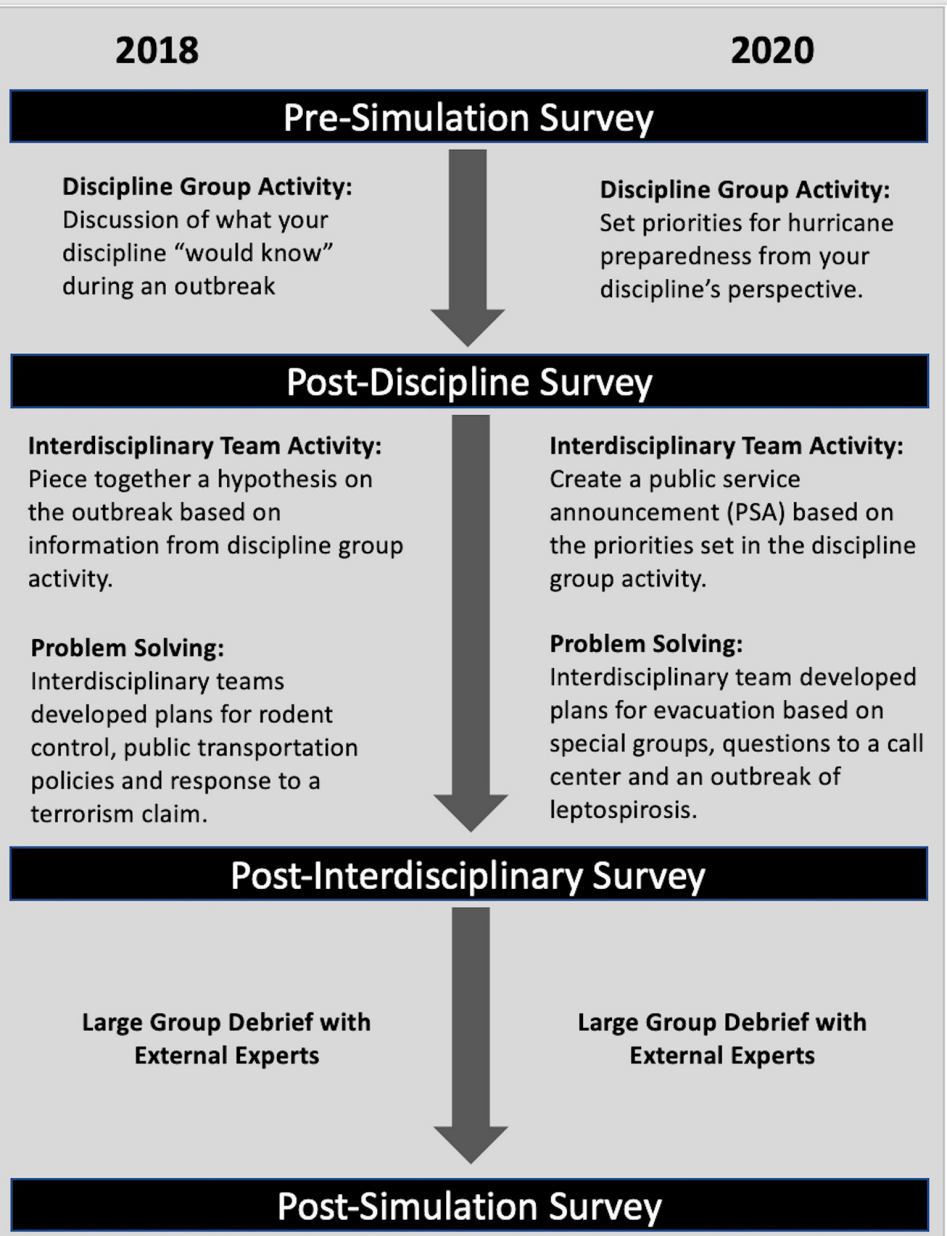
This figure describes the PennDemic framework and the survey points described in this study.

With participation from both institutions, the PennDemic organizing committee developed an innovative framework (Figure 1) for an interprofessional education simulation. The framework structure, based on a jigsaw design, begins with students in discipline groups assigned a specific task before being mixed purposely into interdisciplinary teams (18). In the interdisciplinary team, an activity is completed that requires teams to share findings from their discipline group. This is followed by several problem-solving tasks in the interdisciplinary team and a large group debrief with external, non-academic or “real-world” experts. The organizing committee hypothesized that this framework would increase students' awareness of the importance of interdisciplinary teamwork in disaster preparedness/response.

## Learning Environment

To date, the PennDemic organizing committee has used this framework to develop two scenarios. The first scenario (Simulation 1) focused on a fictitious infectious disease outbreak that took place over several months, although the actual simulation was held on a single day in November 2018. Students were initially provided only with the information that their discipline would be expected to have in the early stages of an outbreak. As an example, veterinary students were given information about animal infections, the human health professional students (i.e., nursing and medicine) were given information about the human symptoms, while the public health students were given information about the results of case interviews and reports of disease. Students developed a list of outstanding questions about the ongoing outbreak, which they shared once placed in interdisciplinary teams. Interdisciplinary teams then developed a hypothesis for how the outbreak had progressed, and they were then asked to work together to develop solutions to three problems that emerged as the outbreak progressed.

Based on the success of the first simulation, a second scenario (simulation 2) was developed around a natural disaster, specifically a hurricane that was approaching the region of the institutions. During the day-long simulation in January 2020, students once again started in their discipline groups. They were asked to prepare a list of key points they would want the public to know for hurricane preparedness from the perspective of their discipline. Once in interdisciplinary teams, students were first asked to develop public service announcements (PSAs) based on agreed upon points from each discipline. Then, as the hurricane approached, students were asked to developed response plans in either an urban or rural setting to several emergency scenarios, such as determining if a specific population should shelter-in-place or evacuate. Both scenarios ran over the course of a single full-day Saturday event.

Both scenarios were specifically designed to emphasize the importance of interdisciplinary collaboration for disaster preparedness/response. Because of the novelty of the approach, including such a broad range of disciplines, we decided to include evaluation as part of each event. Students were asked to complete surveys before the event (pre-simulation), after working in discipline-specific group (post-discipline), after working in interdisciplinary teams (post-interdisciplinary), and then at the end of the event (post-simulation).

## Results

### Study Aim

The aim of this study was to evaluate the effect of a novel framework developed for interprofessional education on student confidence and interest in disaster preparedness/response and interdisciplinary teamwork.

### Processes and Tools

Students applied to participate in the simulation following email distributions through listservs to schools and programs at both institutions. Students were asked to fill out a short application form that included name, degree program, year in degree program, and several short answer items to gauge their previous experience(s) in disaster preparedness/response and their interest in participating in the simulations. To assess the effect of the simulation, students were asked to complete four different surveys. The surveys were anonymous, and students were not required to participate. The University of Pennsylvania IRB determined that the study was for Quality Improvement and thus did not meet the definition of research.

The timing of survey administration is illustrated in Figure 1. The pre-simulation survey included demographic items (degree, year in program, gender, and ethnicity), as well as 10-point-Likert scale items about experience, confidence, interest in disaster preparedness/response and in working on an interprofessional team. In addition, the pre-simulation survey asked one free response question about what first steps that might be taken in the simulation of an epidemic (Simulation 1) or a natural disaster (Simulation 2); because that question was used to get the students thinking about the scenario, the results were not analyzed. The post-simulation survey repeated the 10-point Likert scale items about confidence and interest in disaster preparedness/response and in working on an interprofessional team. There were also 10-point Likert scale items about overall enjoyment, knowledge gained, clarity of goals, quality of the materials provided, and two free response items about what the simulation did well and what could be improved.

Surveys were also administered after the first activity was completed in the discipline group (post-discipline survey) and after the second and third activities were completed in the interdisciplinary teams (post-interdisciplinary survey). These surveys used a 10-point Likert scale to evaluate how well the teams worked: team communication skills, team attitude and the team's reliability. Free response items were used to evaluate what the discipline and interdisciplinary teams did well and what could be improved.

The discipline-specific survey data could not be analyzed because of the small number of students in each discipline. Therefore, the survey data were analyzed as a single group. Confidence and interest pre-scores and post-scores, as well as the experience in a discipline and interdisciplinary teams, were compared using the Student's *t*-test function in Microsoft Excel for Mac (Version 16.44). Answers to the free response items were coded and analyzed using the program Dedoose (19). The short answers were also analyzed for word frequency using WordCounter from DataBasic (20).

### Student Demographics

Simulation 1 had 81 student participants from nine disciplines: biomedical research (21); nursing (15); public health (13; including two dual degree with social work); counseling (9); veterinary medicine (7); medicine (5; including one dual degree with epidemiology); social work (5; including two dual degrees with public health); pharmacy (5); epidemiology (2; including one dual degree with medicine) and social policy students (2). Six of the students were in dual degree programs, therefore the sum of the numbers listed is >81. Additionally, eight of the biomedical research doctoral students were in a public health certificate program. The group included eight undergraduates (all in nursing), 26 first year graduate students, and 47 second year or above graduate students. For the first activity, students were grouped by discipline, each led by a facilitator from their discipline where possible. Note that the two social policy students were included in the group with Master of Social Work students. For the second and third activities, twelve groups were purposely formed to be as interdisciplinary as possible, given the unequal numbers from each discipline: the application materials were used to assign students to disciplines based on past qualifications or experiences. Response rates for the survey were very good, with rates of 89% (pre-survey), 90% (discipline-specific survey), 81% (interprofessional survey), and 80% (post-survey). Gender information was only asked on the pre-survey. Of the group that answered, 86% identified as female and 14% identified as male, a gender imbalance expected given the represented disciplines.

Simulation 2 had a total of 56 students from 14 disciplines, including: biomedical research (10); pharmacy (7); public health (6); social work (6); occupational therapy (4); dentistry (3); design (3); veterinarian medicine (3); epidemiology (2); nursing (2); counseling (2); social policy (2); engineering (2); environmental studies (2); physical therapy (1); anthropology (1, dual degree with medicine), and medicine (1, dual degree with anthropology). One of the students was in a dual degree program, therefore the sum of the numbers listed is >56. Four of the biomedical doctoral students were in a public health certificate program. The group included 4 undergraduates, including 1 in pharmacy, 2 in environmental sciences, and 1 submatriculated into public health), 21 first year graduate students, and 30 second year or above graduate students. Given the small numbers of students for some of the disciplines, the groups for the first activity were assembled as follows: biomedical sciences, clinical, design/environment/engineering, pharmacy, social work/counseling, and public health/non-profit leadership/public policy. For the second and third activities, seven teams were purposely formed to be as interdisciplinary as possible, given the unequal numbers from each discipline. Response rates for the surveys were excellent with rates of 96% (pre-survey), 98% (discipline-specific survey), 98% (interprofessional survey), and 95% (post-survey). Gender information was only asked on the pre-survey. Of the group that answered, 70% identified as female, 22% as male, and 9% gender diverse or preferred not to answer; this gender imbalance was expected given the represented disciplines. Table 1 summarizes student demographics.

**Table 1:** This table describes the demographics of student participants.

	**Simulation 1**	**Simulation 2**
Total number of students	81	56
Number of different disciplines	9	14
% Senior graduate students (2nd year +)	58%	53%
% New graduate students (1st year)	32%	38%
% Female	86%	70%

### Quantitative Analysis of Surveys

Overall, both simulations were rated very highly by students. The average scores in the post-simulation survey for overall enjoyment, knowledge gained, clarity of goals, and quality of materials were >8.5 out of a Likert-scale of 10. There was no statistical difference in the scores between simulations 1 and 2 (data not shown).

Comparison of the pre-simulation and post-simulation surveys showed an increase in average interest scores among students in both disaster preparedness/response and interdisciplinary teamwork (Table 2). Differences in interest scores were found to be significant for both simulations with regards to disaster preparedness/response (Simulation 1, *p* = 0.02; Simulation 2, *p* ≤ 0.01), but only significant for Simulation 1 with regards to interdisciplinary teamwork (Simulation 1, *p* = 0.04; Simulation 2, *p* = 0.08). Confidence levels were also ranked higher by students in disaster preparedness/response and interdisciplinary teamwork. A significant difference was found for confidence levels for both disaster preparedness/response (Simulation 1, *p* ≤ 0.001; Simulation 2, *p* ≤ 0.001) and interdisciplinary teamwork (Simulation 1, *p* ≤ 0.001; Simulation 2, *p* ≤ 0.001). Importantly, there was no statistical difference in the scores between Simulation 1 and Simulation 2 (Table 2; analysis not shown), again suggesting the importance of the overall framework rather than the type of scenario.

**Table 2:** This table compares the self-reported pre-simulation and post simulation interest and confidence scores for disaster preparedness/response and working on an interdisciplinary team by iteration.

**Scale**	**Pre-simulation**	**Post-simulation**	***p***
	**(std dev)**	**(std dev)**	
**Simulation 1 (2018)**			
Preparedness confidence	3.82 (2.32)	7.12 (1.48)	<0.001
Preparedness interest	8.75 (1.37)	9.26 (1.25)	0.02
Teamwork confidence	6.67 (2.16)	8.63 (1.32)	<0.001
Teamwork interest	8.90 (1.68)	9.42 (1.20)	0.04
**Simulation 2 (2020)**			
Preparedness confidence	3.85 (1.88)	7.60 (1.30)	<0.001
Preparedness interest	8.39 (1.50)	9.12 (1.02)	<0.01
Teamwork confidence	6.96 (2.16)	8.77 (1.35)	<0.001
Teamwork interest	8.80 (1.68)	9.31 (1.25)	0.08

On average, students ranked the scores of overall team effectiveness and positive group behaviors (good communication, positive attitudes, and reliability) higher for the interdisciplinary team than their discipline specific groups (Table 3). These differences were statistically significant for Simulation 1, but not Simulation 2. All average scores were high, with 8.37 as the lowest score.

**Table 3:** This table compares the self-reported experiences of students in their discipline and interdisciplinary groups for overall and specific positive group behaviors.

**Scale**	**Discipline**	**Interdisciplinary**	***p***
	**(std dev)**	**(std dev)**	
**Simulation 1 (2018)**			
How well did the group work?	8.37 (1.43)	9.23 (0.91)	<0.001
Good communication skills?	8.46 (1.60)	9.17 (0.97)	<0.001
Positive attitude?	9.17 (1.15)	9.5 (0.62)	0.04
Reliability?	8.63 (2.72)	9.28 (1.33)	0.01
**Simulation 2 (2020)**			
How well did the group work?	8.85 (1.18)	9.15 (1.07)	0.16
Good communication skills?	8.69 (1.35)	8.98 (1.31)	0.25
Positive attitude?	9.09 (1.16)	9.25 (0.99)	0.42
Reliability?	8.76 (1.20)	9.04 (1.19)	0.23

### Qualitative Analysis of Surveys

Analysis of the short answer items in the discipline-specific and interdisciplinary surveys showed similarity between Simulation 1 and 2. For the question “what did your discipline-specific group do well,” the most common answers for both Simulation 1 and Simulation 2 had the following themes: sharing and working together, communications, and brainstorming. One response from Simulation 1 touched on all of this: “Sharing information, asking questions of one another, collaborating to determine what next information we all wanted to know.” For the question “what did your interdisciplinary group do well,” the answers for both Simulation 1 and Simulation 2 include the themes from the discipline-specific answers, as well as a new one: contributions of the disciplines. One response from Simulation 2 touched on all of this: “Our conversations effectively integrated all discipline-specific perspectives and all voices, making for a truly collaborative environment.”

For the question “what could your discipline-specific group improve,” the themes centered on working together: sharing information and personal interactions (either students who didn't talk or those that spoke too much) for Simulation 1 and focus, organization, and personal interactions for Simulation 2. For “what could your interdisciplinary group improve,” the themes were organization and personal interactions for Simulation 1 and time management for Simulation 2. Interestingly for both Simulation 1 and Simulation 2, the answers for interdisciplinary group improvement included a logistical theme: missing a discipline. This was particularly true for Simulation 2, where the small numbers of students per discipline meant that most teams were missing representation of at least one, if not two disciplines. Another interesting theme for interdisciplinary team improvement was the answer “nothing” or “N/A,” indicating that the interdisciplinary team worked well. This theme was particularly prevalent for Simulation 1, comprising over a third of the answers.

The post-simulation survey asked two open-ended items: “what did PennDemic do well” and “what could PennDemic improve.” The most common theme for what the PennDecmic simulation did well was centered around the interdisciplinary aspect of the simulation, with several answers expanding on the strength of going from discipline-specific to interdisciplinary. This answer was found in 50 and 40% of answers from Simulation 1 and 2, respectively, a higher prevalence than any other theme for any short answers. An example of some of these answers to this question can be found in Table 4. The themes for what simulation could improve were mostly centered on logistics, from overall timing of the simulation and noise levels of the room (Simulation 1) to missing disciplines (Simulation 1 and 2), with additional suggestions on how to improve the specific activities within the scenario (Simulations 1 and 2). Still one of the most common themes for both was “nothing” or “N/A,” suggesting that the respondents thought that the simulations went well.

**Table 4:** This table contains examples of comments made by students about the simulation.

**What did Simulation do well?**
**Simulation 1 (2018)**
“I really liked how it showed the numerous disciplines that affect and are impacted by health. We all play a role.”
“I loved working across disciplines. I think I gained a lot of knowledge from my colleagues.”
“I think the interdisciplinary focus was important and helped inform my understanding of the outbreak.”
**Simulation 2 (2020)**
“I now realize how many people with unique backgrounds need to be involved in order to keep people safe.”
“I enjoyed the balance between the conversation in the disciplinary groups and in the interprofessional group.”
“Genius idea to bring people from different background/professional aspects to work together toward the same goal”

## Discussion

The organizers of the PennDemic set out to develop a sustainable model to build interdisciplinary, disaster preparedness training simulations at the University of Pennsylvania and a neighboring institution, the University of the Sciences. They developed a novel framework that was applied to two different scenarios: an infectious disease outbreak (Simulation 1) and a hurricane (Simulation 2). Both simulations were highly rated in post-simulation survey items on overall enjoyment, knowledge gained, clarity of goals, and quality of materials, with no statistical difference between the two simulations, suggesting that the type of disaster (infectious disease vs. natural disaster) was not relevant to the implementation of the framework.

Comparison of pre-simulation and post-simulation surveys from both simulations shows that the PennDemic structure is an effective approach to build student confidence and interest in both disaster preparedness/response and interdisciplinary teamwork (Table 2). It's important to note that these activities were not required as part of a degree program. Instead, students opted in to participate on a Saturday. Based on the applications, we hypothesized that the students had a higher than average interest in disaster preparedness and teamwork, which makes the post-simulation increase in both interest and confidence even more significant. The average scores for interest and confidence in both disaster preparedness/response and interdisciplinary teamwork were statistically similar for the pre-simulation and post-simulation surveys for both Simulations 1 and 2 (Table 2; analysis not shown). This again supports the idea that the framework of the simulation, and not the topics, played a more significant role in generating interest and confidence in both disaster preparedness/response and interdisciplinary teamwork.

The average confidence scores in disaster preparedness/response and interdisciplinary teamwork were significantly higher post-simulation for both scenarios. This quantitative analysis is supported by the similarity in answers between Simulation 1 and Simulation 2 as to what went well and what could be improved. The disaster preparedness/response average scores were lower than those for interdisciplinary teamwork, a result supported by the responses to the post-simulation question about what PennDemic did well, where the emphasis was on the interdisciplinary nature of the activity. The increase in scores was larger for disaster preparedness/response than interdisciplinary teamwork, suggesting that students felt that they did learn about disaster preparation.

The average interest scores in disaster preparedness/response were significantly elevated following the simulation for both scenarios. Post-simulation interdisciplinary team interest scores were also increased, but only statistically significantly different for Simulation 1. This may be explained by the mix of students in Simulation 2, with fewer students but a wider range of disciplines (Table 1). The organizers had deliberately increased the distribution of the application for Simulation 2, explaining the breadth of disciplines. The smaller number of students was attributed to scheduling issues within the semester, an unseasonably cold day, and (ironically) a flu outbreak, which caused last-minute cancellations. It is important to note that the average scores for interest in disaster preparedness/response and interdisciplinary teamwork started out high in the pre-simulation survey, likely biased by the types of students who would participate in this extra-curricular simulation.

Given the “jigsaw design” where student groups were rearranged from discipline groups to interdisciplinary teams during the simulation, we also compared their experiences in the two different groups (Table 3). A rise in average rankings for the question “How well did your group work?” was seen in both Simulations 1 and 2 from discipline groups to interdisciplinary teams. Additionally, higher average rankings of positive behaviors (good communication, positive attitude, and reliability) were also found for the interdisciplinary teams. This increase suggests that students took lessons learned in their discipline-specific group and used that to work effectively in an interdisciplinary team.

Although the values for group measures (communication, attitude, and reliability) increased between discipline and interdisciplinary teams in both scenarios, the differences were only statistically significant in Simulation 1 and not Simulation 2. There are two possible reasons for this. First, Simulation 2 had lower participation numbers and more disciplines, forcing the discipline groups to be more broadly defined into roles (e.g., clinical [included medical, dental, nursing, pharmacy, and veterinary students], environmental [included urban design, engineering, environmental studies]) than Simulation 1 (e.g., veterinary, public health, nursing, pharmacy, social work). This may suggest that the perceived differences in the team's teamwork is more noticeable when moving from a narrower field. Second, the lack of statistically significant findings in Simulation 2 could be due to differences in the type of specific activities completed throughout the day. Regardless, the average scores (Table 3) were quite high, suggesting that the students felt that both their discipline and interdisciplinary teams did quite well.

An important take home point of the PennDemic framework is careful composition of both discipline and interdisciplinary teams. In survey items on what your interdisciplinary team could have improved, one of the most common answers concerned the lack of a particular discipline. This showed that students recognized the need for broad representation during disaster and emergency preparedness situations.

The broader implications for pedagogy should be considered for fields that are commonly included in IPE exercises and for those that are not traditionally included. A more inclusive framework of IPE such as PennDemic furthers training for medical, nursing, and pharmacy students and prepares them for collaboration outside of traditional fields where common terminology, goals and experiences may vary greatly. Fields such as public health and veterinary medicine are often not included in IPE activities, but modern curricular requirements highlight a need for knowledge, skills, and attitudes that are honed by participation in IPE activities. The jigsaw framework that we developed advances pedagogical innovation by being accessible to any number of disciplines. For example, in veterinary medicine, an entire national competency domain of “collaboration” has recently been established in order to build skills that help veterinarians work with “diverse colleagues, clients and other stakeholders and demonstrates skills as a leader and inter-professional team member to improve outcomes and reduce error” (21). For students in fields like biomedical sciences or engineering, PennDemic gives them exposure to the broader implications of their work. For all students, the PennDemic framework allows participants the opportunity to demonstrate expertise from within their own field and then integrate their knowledge with what they learn from the expertise of participants from other fields. Limited sample sizes within the different disciplines prevented subgroup analyses, but continued PennDemic iterations will help us investigate the implications for specific fields.

Overall, both PennDemic simulations effectively increased student confidence and interest in both disaster preparedness/response and interdisciplinary teamwork, and also increased awareness of the importance of interdisciplinary teams beyond the traditional healthcare focus. Critical to the success of these simulations, regardless of topic, is the PennDemic framework, with its transition from discipline-specific work to interdisciplinary teamwork and problem solving and ending with a large group debrief. This framework will be used in future iterations of simulations for a variety of other potential disasters such as other types of infectious diseases, as well as chemical or radiological disasters. The use of the PennDemic framework is helpful to engage those in fields often excluded from interprofessional education. The framework can be used outside of traditional educational settings to inspire formation of interdisciplinary relationships to work together in disaster preparedness.

The authors encourage educators to design their own simulations using the PennDemic framework based on the jigsaw and problem-solving model described in this manuscript. This approach allows for implementation of appropriate scenarios and simulations which can be targeted to the specific disciplines represented at an individual institution. Given that a large number of permutations of different disciplines could be possible, the use of our specific topics may or may not be appropriate. There are also geographic specific portions of the simulations that may not be appropriate for all institutions. For groups interested in use of the simulation materials described in this study an electronic request can be made for materials at www.vet.upenn.edu/penndemic-interest-form after agreement for appropriate attribution.

## Acknowledgment of Any Conceptual, Methodological, Environmental, or Material Constraints

Disaster preparedness/response is an incredibly complex field; therefore, it is not possible to engage students in all aspects with the time constraints of a single day event. Careful attention should be paid to topics covered. Another constraint is the need to recruit a sufficient number of students from different disciplines to evenly divide them among groups, therefore other groups with the desire to implement a similar simulation should tailor their simulations to fit the student population available or provide materials if certain groups are missing students from a discipline. In this study, long-term impacts of the simulation in disaster preparedness/response interest could not be assessed, but it is likely that experience in PennDemic may motivate students to seek out future opportunities to further explore relevant career pathways within their own fields. Regardless of these constraints, the data presented in this report suggests that the PennDemic framework can be used to engage students from a variety of backgrounds to build interest in confidence in disaster preparedness/response and interdisciplinary teamwork.

## Data Availability Statement

The raw data supporting the conclusions of this article will be made available by the authors, without undue reservation.

## Author Contributions

SC, BJ, SR, and DB developed the Concept for PennDemic. DB lead funding efforts and developed surveys. SC and HN drafted manuscript. HN performed statistical and qualitative analyses. All authors contributed to the design and implementation of the PennDemic simulations and edited the manuscript.

## Conflict of Interest

The authors declare that the research was conducted in the absence of any commercial or financial relationships that could be construed as a potential conflict of interest.
